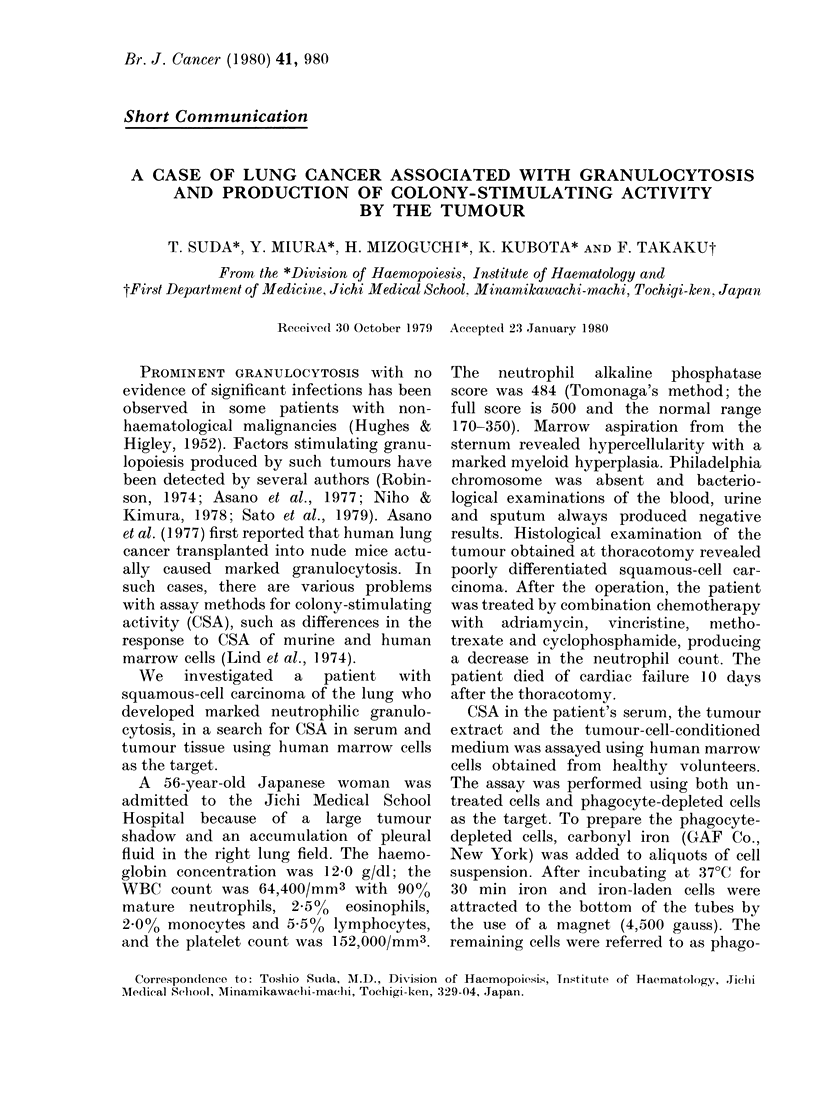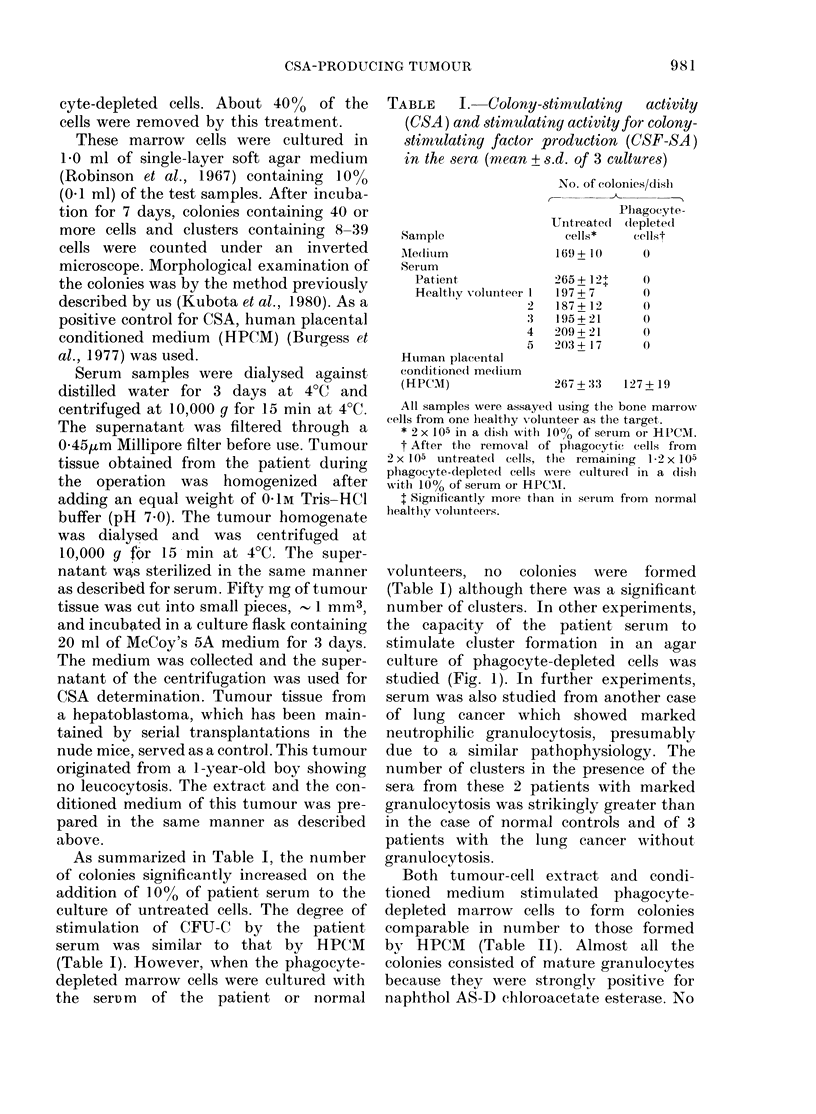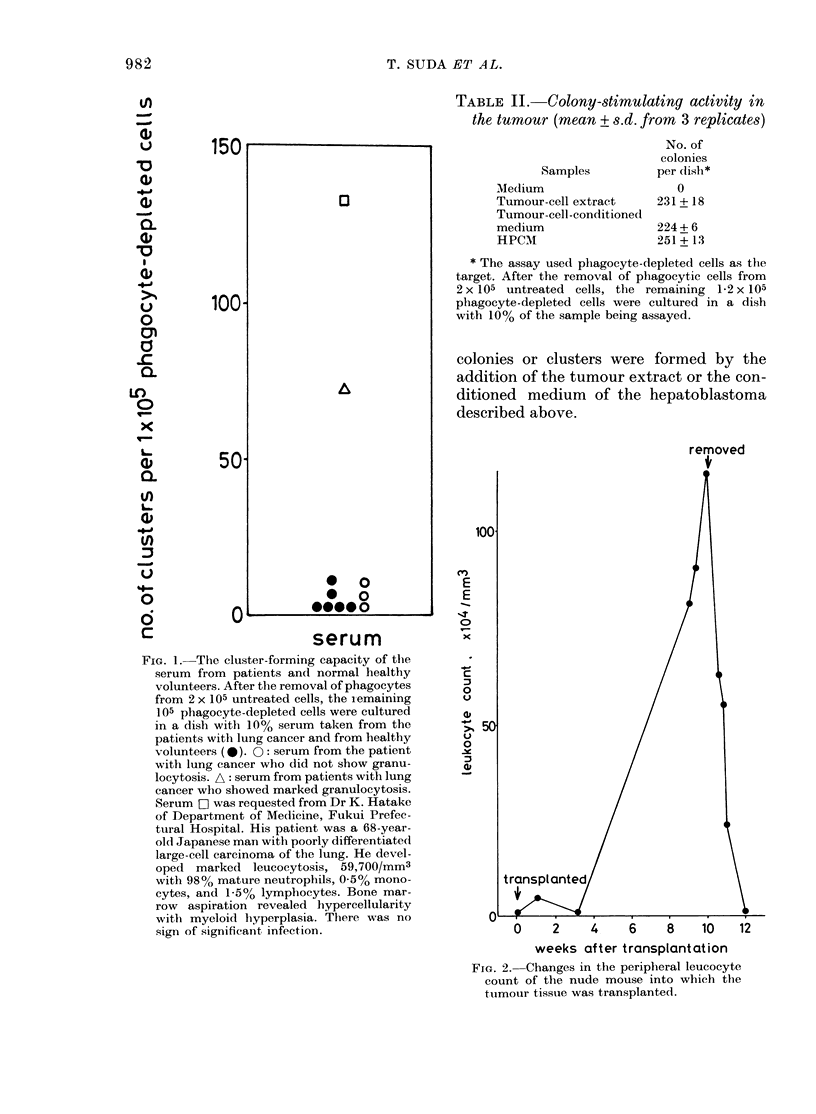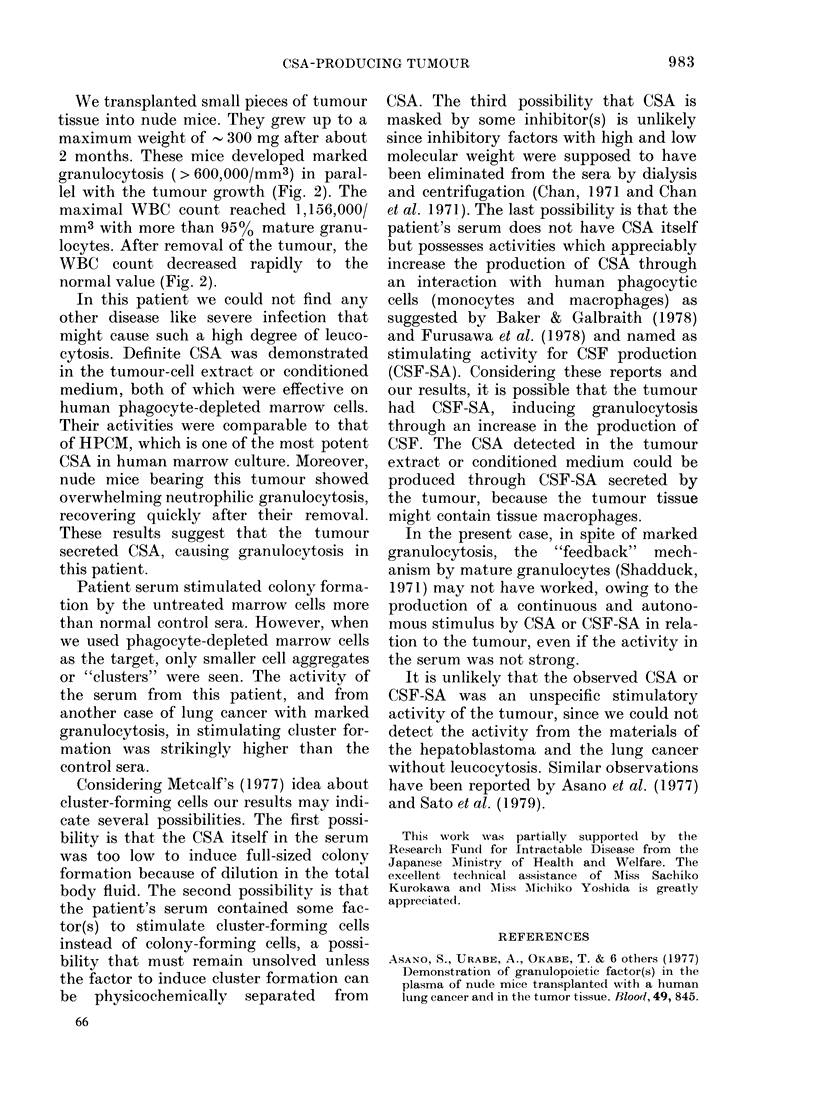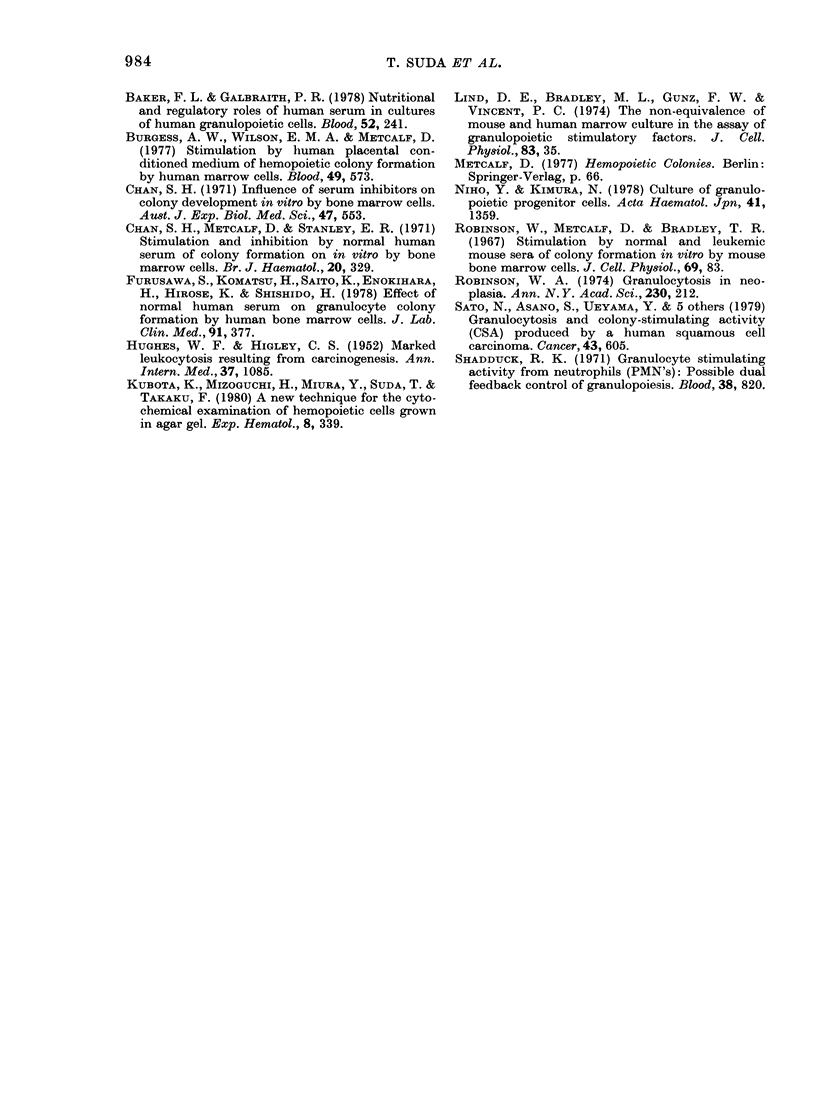# A case of lung cancer associated with granulocytosis and production of colony-stimulating activity by the tumour.

**DOI:** 10.1038/bjc.1980.177

**Published:** 1980-06

**Authors:** T. Suda, Y. Miura, H. Mizoguchi, K. Kubota, F. Takaku


*Br. J. Cancer* (1980) **41**, 980

***Short Communication***

### A CASE OF LUNG CANCER ASSOCIATED WITH GRANULOCYTOSIS AND PRODUCTION OF COLONY-STIMULATING ACTIVITY BY THE TUMOUR

T. SUDA*, Y. MIURA*, H. MIZOGUCHI*, K. KUBOTA* AND F. TAKAKU†

*From the *Division of Haemopoiesis, Institute of Haematology and †First Department of Medicine, Jichi Medical School, Minamikawachi-machi, Tochigi-ken, Japan*

Received 30 October 1979 Accepted 23 January 1980

PROMINENT GRANULOCYTOSIS with no evidence of significant infections has been observed in some patients with non-haematological malignancies (Hughes & Higley, 1952). Factors stimulating granulopoiesis produced by such tumours have been detected by several authors (Robinson, 1974; Asano *et al.*, 1977; Niho & Kimura, 1978; Sato *et al.*, 1979). Asano *et al.* (1977) first reported that human lung cancer transplanted into nude mice actually caused marked granulocytosis. In such cases, there are various problems with assay methods for colony-stimulating activity (CSA), such as differences in the response to CSA of murine and human marrow cells (Lind *et al.*, 1974).

We investigated a patient with squamous-cell carcinoma of the lung who developed marked neutrophilic granulocytosis, in a search for CSA in serum and tumour tissue using human marrow cells as the target.

A 56-year-old Japanese woman was admitted to the Jichi Medical School Hospital because of a large tumour shadow and an accumulation of pleural fluid in the right lung field. The haemoglobin concentration was 12·0 g/dl; the WBC count was 64,400/mm$^3$ with 90% mature neutrophils, 2·5% eosinophils, 2·0% monocytes and 5·5% lymphocytes, and the platelet count was 152,000/mm$^3$. The neutrophil alkaline phosphatase score was 484 (Tomonaga's method; the full score is 500 and the normal range 170–350). Marrow aspiration from the sternum revealed hypercellularity with a marked myeloid hyperplasia. Philadelphia chromosome was absent and bacteriological examinations of the blood, urine and sputum always produced negative results. Histological examination of the tumour obtained at thoracotomy revealed poorly differentiated squamous-cell carcinoma. After the operation, the patient was treated by combination chemotherapy with adriamycin, vincristine, methotrexate and cyclophosphamide, producing a decrease in the neutrophil count. The patient died of cardiac failure 10 days after the thoracotomy.

CSA in the patient's serum, the tumour extract and the tumour-cell-conditioned medium was assayed using human marrow cells obtained from healthy volunteers. The assay was performed using both untreated cells and phagocyte-depleted cells as the target. To prepare the phagocyte-depleted cells, carbonyl iron (GAF Co., New York) was added to aliquots of cell suspension. After incubating at 37°C for 30 min iron and iron-laden cells were attracted to the bottom of the tubes by the use of a magnet (4,500 gauss). The remaining cells were referred to as phago-

Correspondence to: Toshio Suda, M.D., Division of Haemopoiesis, Institute of Haematology, Jichi Medical School, Minamikawachi-machi, Tochigi-ken, 329-04, Japan.

cyte-depleted cells. About 40% of the cells were removed by this treatment.

These marrow cells were cultured in 1·0 ml of single-layer soft agar medium (Robinson *et al.*, 1967) containing 10% (0·1 ml) of the test samples. After incubation for 7 days, colonies containing 40 or more cells and clusters containing 8–39 cells were counted under an inverted microscope. Morphological examination of the colonies was by the method previously described by us (Kubota *et al.*, 1980). As a positive control for CSA, human placental conditioned medium (HPCM) (Burgess *et al.*, 1977) was used.

Serum samples were dialysed against distilled water for 3 days at 4°C and centrifuged at 10,000 *g* for 15 min at 4°C. The supernatant was filtered through a 0·45μm Millipore filter before use. Tumour tissue obtained from the patient during the operation was homogenized after adding an equal weight of 0·1M Tris–HCl buffer (pH 7·0). The tumour homogenate was dialysed and was centrifuged at 10,000 *g* for 15 min at 4°C. The supernatant was sterilized in the same manner as described for serum. Fifty mg of tumour tissue was cut into small pieces, ~1 mm³, and incubated in a culture flask containing 20 ml of McCoy's 5A medium for 3 days. The medium was collected and the supernatant of the centrifugation was used for CSA determination. Tumour tissue from a hepatoblastoma, which has been maintained by serial transplantations in the nude mice, served as a control. This tumour originated from a 1-year-old boy showing no leucocytosis. The extract and the conditioned medium of this tumour was prepared in the same manner as described above.

As summarized in Table I, the number of colonies significantly increased on the addition of 10% of patient serum to the culture of untreated cells. The degree of stimulation of CFU-C by the patient serum was similar to that by HPCM (Table I). However, when the phagocyte-depleted marrow cells were cultured with the serum of the patient or normal volunteers, no colonies were formed (Table I) although there was a significant number of clusters. In other experiments, the capacity of the patient serum to stimulate cluster formation in an agar culture of phagocyte-depleted cells was studied (Fig. 1). In further experiments, serum was also studied from another case of lung cancer which showed marked neutrophilic granulocytosis, presumably due to a similar pathophysiology. The number of clusters in the presence of the sera from these 2 patients with marked granulocytosis was strikingly greater than in the case of normal controls and of 3 patients with the lung cancer without granulocytosis.

Both tumour-cell extract and conditioned medium stimulated phagocyte-depleted marrow cells to form colonies comparable in number to those formed by HPCM (Table II). Almost all the colonies consisted of mature granulocytes because they were strongly positive for naphthol AS-D chloroacetate esterase. No

TABLE I.—*Colony-stimulating activity (CSA) and stimulating activity for colony-stimulating factor production (CSF-SA) in the sera (mean ± s.d. of 3 cultures)*

| Sample | No. of colonies/dish: Untreated cells* | Phagocyte-depleted cells† |
|---|---|---|
| Medium | 169 ± 10 | 0 |
| Serum | | |
| Patient | 265 ± 12‡ | 0 |
| Healthy volunteer 1 | 197 ± 7 | 0 |
| 2 | 187 ± 12 | 0 |
| 3 | 195 ± 21 | 0 |
| 4 | 209 ± 21 | 0 |
| 5 | 203 ± 17 | 0 |
| Human placental conditioned medium (HPCM) | 267 ± 33 | 127 ± 19 |

All samples were assayed using the bone marrow cells from one healthy volunteer as the target.

* $2 \times 10^5$ in a dish with 10% of serum or HPCM.

† After the removal of phagocytic cells from $2 \times 10^5$ untreated cells, the remaining $1{\cdot}2 \times 10^5$ phagocyte-depleted cells were cultured in a dish with 10% of serum or HPCM.

‡ Significantly more than in serum from normal healthy volunteers.

FIG. 1.—The cluster-forming capacity of the serum from patients and normal healthy volunteers. After the removal of phagocytes from $2 \times 10^5$ untreated cells, the remaining $10^5$ phagocyte-depleted cells were cultured in a dish with 10% serum taken from the patients with lung cancer and from healthy volunteers (●). ○ : serum from the patient with lung cancer who did not show granulocytosis. △ : serum from patients with lung cancer who showed marked granulocytosis. Serum □ was requested from Dr K. Hatake of Department of Medicine, Fukui Prefectural Hospital. His patient was a 68-year-old Japanese man with poorly differentiated large-cell carcinoma of the lung. He developed marked leucocytosis, 59,700/mm³ with 98% mature neutrophils, 0·5% monocytes, and 1·5% lymphocytes. Bone marrow aspiration revealed hypercellularity with myeloid hyperplasia. There was no sign of significant infection.

TABLE II.—*Colony-stimulating activity in the tumour (mean ± s.d. from 3 replicates)*

| Samples | No. of colonies per dish* |
|---|---|
| Medium | 0 |
| Tumour-cell extract | 231 ± 18 |
| Tumour-cell-conditioned medium | 224 ± 6 |
| HPCM | 251 ± 13 |

* The assay used phagocyte-depleted cells as the target. After the removal of phagocytic cells from $2 \times 10^5$ untreated cells, the remaining $1{\cdot}2 \times 10^5$ phagocyte-depleted cells were cultured in a dish with 10% of the sample being assayed.

colonies or clusters were formed by the addition of the tumour extract or the conditioned medium of the hepatoblastoma described above.

FIG. 2.—Changes in the peripheral leucocyte count of the nude mouse into which the tumour tissue was transplanted.

We transplanted small pieces of tumour tissue into nude mice. They grew up to a maximum weight of $\sim 300$ mg after about 2 months. These mice developed marked granulocytosis ($> 600{,}000/mm^3$) in parallel with the tumour growth (Fig. 2). The maximal WBC count reached $1{,}156{,}000/mm^3$ with more than 95% mature granulocytes. After removal of the tumour, the WBC count decreased rapidly to the normal value (Fig. 2).

In this patient we could not find any other disease like severe infection that might cause such a high degree of leucocytosis. Definite CSA was demonstrated in the tumour-cell extract or conditioned medium, both of which were effective on human phagocyte-depleted marrow cells. Their activities were comparable to that of HPCM, which is one of the most potent CSA in human marrow culture. Moreover, nude mice bearing this tumour showed overwhelming neutrophilic granulocytosis, recovering quickly after their removal. These results suggest that the tumour secreted CSA, causing granulocytosis in this patient.

Patient serum stimulated colony formation by the untreated marrow cells more than normal control sera. However, when we used phagocyte-depleted marrow cells as the target, only smaller cell aggregates or "clusters" were seen. The activity of the serum from this patient, and from another case of lung cancer with marked granulocytosis, in stimulating cluster formation was strikingly higher than the control sera.

Considering Metcalf's (1977) idea about cluster-forming cells our results may indicate several possibilities. The first possibility is that the CSA itself in the serum was too low to induce full-sized colony formation because of dilution in the total body fluid. The second possibility is that the patient's serum contained some factor(s) to stimulate cluster-forming cells instead of colony-forming cells, a possibility that must remain unsolved unless the factor to induce cluster formation can be physicochemically separated from CSA. The third possibility that CSA is masked by some inhibitor(s) is unlikely since inhibitory factors with high and low molecular weight were supposed to have been eliminated from the sera by dialysis and centrifugation (Chan, 1971 and Chan *et al.* 1971). The last possibility is that the patient's serum does not have CSA itself but possesses activities which appreciably increase the production of CSA through an interaction with human phagocytic cells (monocytes and macrophages) as suggested by Baker & Galbraith (1978) and Furusawa *et al.* (1978) and named as stimulating activity for CSF production (CSF-SA). Considering these reports and our results, it is possible that the tumour had CSF-SA, inducing granulocytosis through an increase in the production of CSF. The CSA detected in the tumour extract or conditioned medium could be produced through CSF-SA secreted by the tumour, because the tumour tissue might contain tissue macrophages.

In the present case, in spite of marked granulocytosis, the "feedback" mechanism by mature granulocytes (Shadduck, 1971) may not have worked, owing to the production of a continuous and autonomous stimulus by CSA or CSF-SA in relation to the tumour, even if the activity in the serum was not strong.

It is unlikely that the observed CSA or CSF-SA was an unspecific stimulatory activity of the tumour, since we could not detect the activity from the materials of the hepatoblastoma and the lung cancer without leucocytosis. Similar observations have been reported by Asano *et al.* (1977) and Sato *et al.* (1979).

This work was partially supported by the Research Fund for Intractable Disease from the Japanese Ministry of Health and Welfare. The excellent technical assistance of Miss Sachiko Kurokawa and Miss Michiko Yoshida is greatly appreciated.

### REFERENCES

ASANO, S., URABE, A., OKABE, T. & 6 others (1977) Demonstration of granulopoietic factor(s) in the plasma of nude mice transplanted with a human lung cancer and in the tumor tissue. *Blood*, **49**, 845.

BAKER, F. L. & GALBRAITH, P. R. (1978) Nutritional and regulatory roles of human serum in cultures of human granulopoietic cells. *Blood*, **52**, 241.

BURGESS, A. W., WILSON, E. M. A. & METCALF, D. (1977) Stimulation by human placental conditioned medium of hemopoietic colony formation by human marrow cells. *Blood*, **49**, 573.

CHAN, S. H. (1971) Influence of serum inhibitors on colony development *in vitro* by bone marrow cells. *Aust. J. Exp. Biol. Med. Sci.*, **47**, 553.

CHAN, S. H., METCALF, D. & STANLEY, E. R. (1971) Stimulation and inhibition by normal human serum of colony formation on *in vitro* by bone marrow cells. *Br. J. Haematol.*, **20**, 329.

FURUSAWA, S., KOMATSU, H., SAITO, K., ENOKIHARA, H., HIROSE, K. & SHISHIDO, H. (1978) Effect of normal human serum on granulocyte colony formation by human bone marrow cells. *J. Lab. Clin. Med.*, **91**, 377.

HUGHES, W. F. & HIGLEY, C. S. (1952) Marked leukocytosis resulting from carcinogenesis. *Ann. Intern. Med.*, **37**, 1085.

KUBOTA, K., MIZOGUCHI, H., MIURA, Y., SUDA, T. & TAKAKU, F. (1980) A new technique for the cytochemical examination of hemopoietic cells grown in agar gel. *Exp. Hematol.*, **8**, 339.

LIND, D. E., BRADLEY, M. L., GUNZ, F. W. & VINCENT, P. C. (1974) The non-equivalence of mouse and human marrow culture in the assay of granulopoietic stimulatory factors. *J. Cell. Physiol.*, **83**, 35.

METCALF, D. (1977) *Hemopoietic Colonies*. Berlin: Springer-Verlag, p. 66.

NIHO, Y. & KIMURA, N. (1978) Culture of granulopoietic progenitor cells. *Acta Haematol. Jpn*, **41**, 1359.

ROBINSON, W., METCALF, D. & BRADLEY, T. R. (1967) Stimulation by normal and leukemic mouse sera of colony formation *in vitro* by mouse bone marrow cells. *J. Cell. Physiol.*, **69**, 83.

ROBINSON, W. A. (1974) Granulocytosis in neoplasia. *Ann. N.Y. Acad. Sci.*, **230**, 212.

SATO, N., ASANO, S., UEYAMA, Y. & 5 others (1979) Granulocytosis and colony-stimulating activity (CSA) produced by a human squamous cell carcinoma. *Cancer*, **43**, 605.

SHADDUCK, R. K. (1971) Granulocyte stimulating activity from neutrophils (PMN's): Possible dual feedback control of granulopoiesis. *Blood*, **38**, 820.